# Supplementary material for: The ribosome inhibitor chloramphenicol induces motility deficits in human spermatozoa: A proteomic approach identifies potentially involved proteins
Source: Front Cell Dev Biol. 2022 Sep 2;10:965076. doi: 10.3389/fcell.2022.965076 (PMC9478589; doi:10.3389/fcell.2022.965076)
Supplement: Supplementary file 6 [file DataSheet1.DOCX]

Supplementary Material

# Supplementary Figures


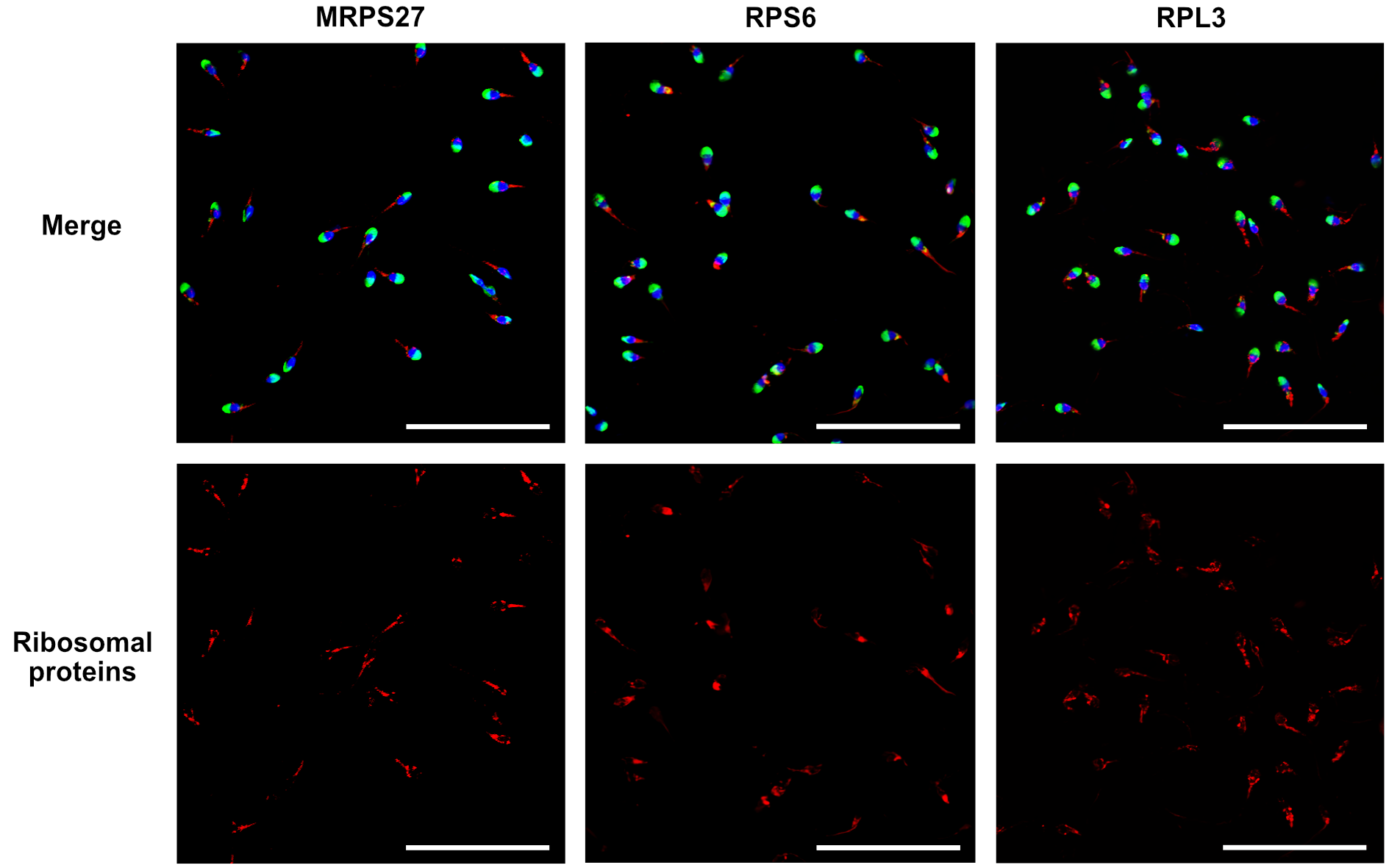


**Figure S1**. General views for the localization of mitochondrial (MRPS27) and cytoplasmic (RPS6 and RPL3) ribosomal proteins in human spermatozoa. Purified human spermatozoa were fixed with 4% paraformaldehyde, permeabilized with 0.3% Triton-X-100 and stained with anti-MRPS27, -RPS6, or-RPL3 (antibodies. Red: Ribosomal proteins, blue: DAPI staining of the nucleus, green: PSA-FITC staining of the acrosome. Scale bar: 50 μm. Representative results of N = 3 experiments.


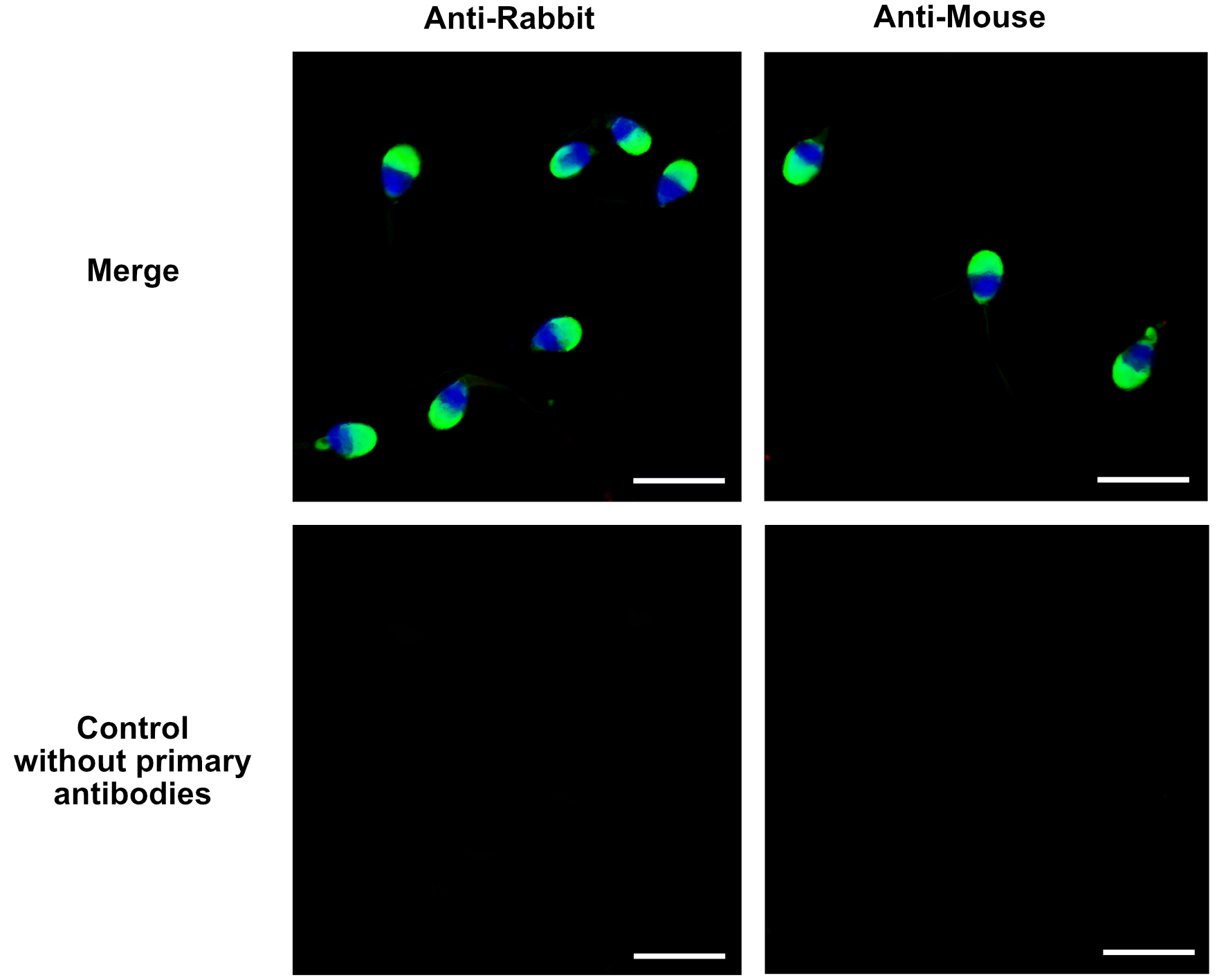


**Figure S2.** Negative controls for the localization of mitochondrial and cytoplasmic ribosomal proteins in human spermatozoa. Purified human spermatozoa were fixed with 4% paraformaldehyde, permeabilized with 0.3% Triton-X-100 and directly incubated with Alexa fluor 568-conjugated goat anti-rabbit or anti-mouse antibodies. Blue: DAPI staining of the nucleus, green: PSA-FITC staining of the acrosome. Scale bar: 10 μm. Representative results of N = 3 experiments.


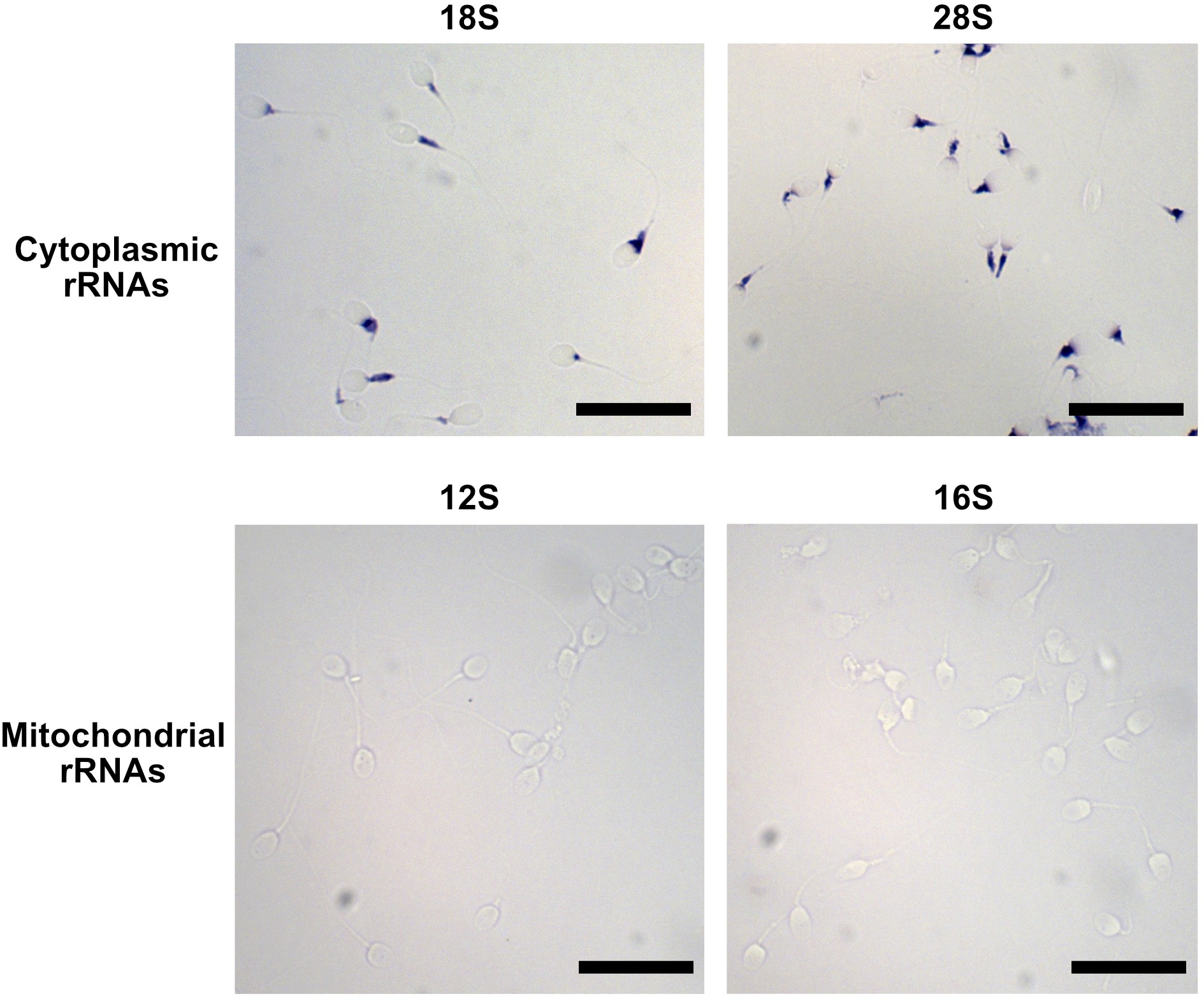


**Figure S3**. General views for the localization of rRNAs in human spermatozoa by *in situ* hybridization. Spermatozoa were labelled using antisense RNA probes targeting 28S, 18S, 16S, and 12S rRNAs. Scale bar: 10 µm.


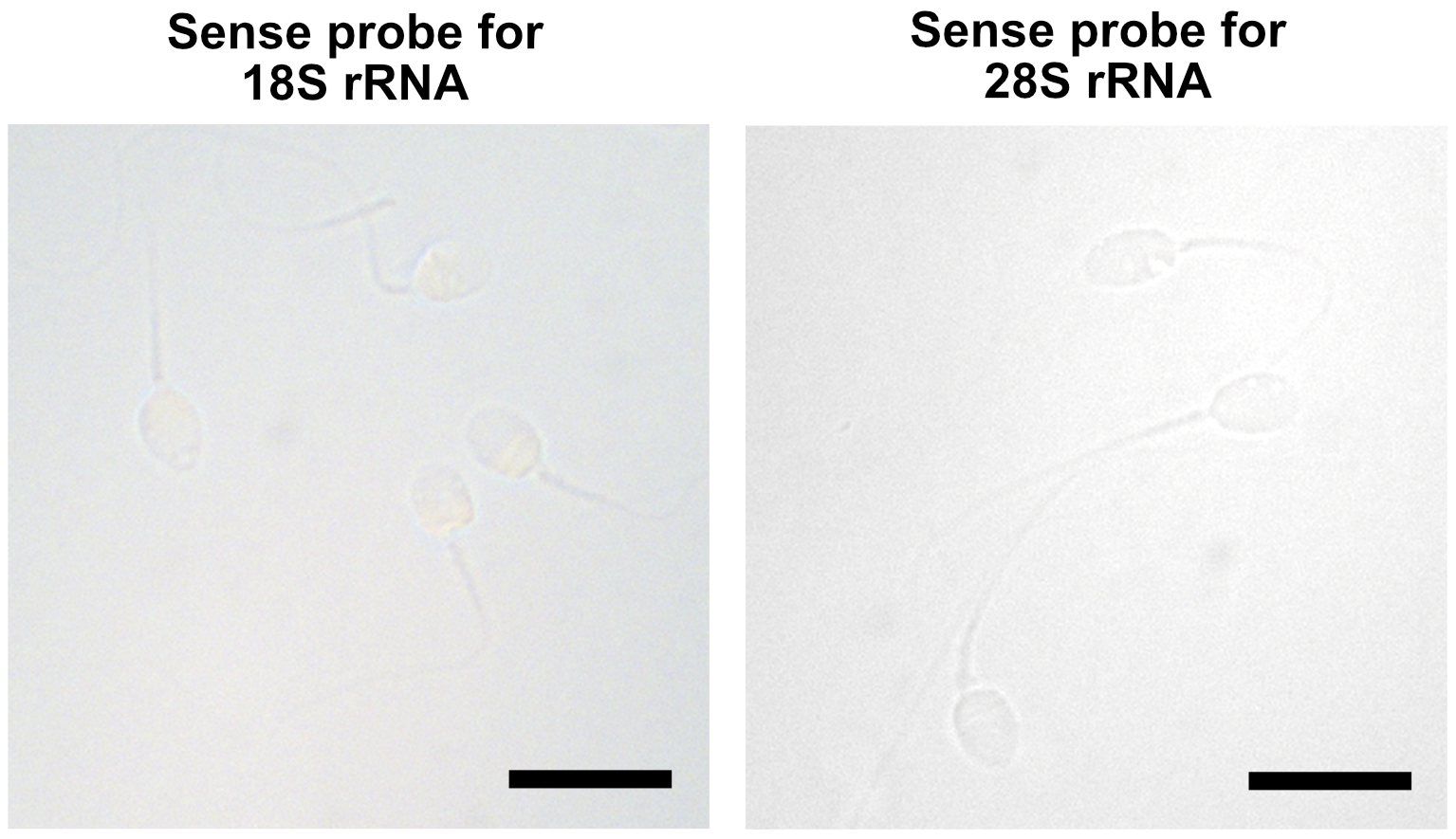


**Figure S4**. Negative controls for the localization of 18S and 28S rRNAs in human spermatozoa by *in situ* hybridization. Spermatozoa were incubated with sense RNA probes for 18S and 28S rRNAs. Scale bar: 10 µm.

# Supplementary Tables

**Table S1.** Ribosomal proteins in published human sperm proteomes 🡪 Excel file

**Table S2.** Primers used to synthesize probes for *in situ* hybridization

| Targeted RNA | Primers | Probe length (bp) |
| --- | --- | --- |
| 28S | **CATTTAGGTGACACTATAGAAG**GCAGGAGGTGTCAGAAAAGTTACC **GGATCCTAATACGACTCACTATAGG**TATTAGTGGGTGAACAATCCAACG | 150 |
| 18S | **CATTTAGGTGACACTATAGAAG**ACGATCAGATACCGTCGTAGTTCC **GGATCCTAATACGACTCACTATAGG**CCTTTAAGTTTCAGCTTTGCAACC | 147 |
| 16S | **GGATCCTAATACGACTCACTATAGG**TGCAGAAGGTATAGGGGTTAGTCC | 151 |
| 12S | **GGATCCTAATACGACTCACTATAGG**GGGGTTTATCGATTACAGAACAGG | 157 |
| In blue: promoter for the T7 RNA polymerase | |  |
| In red: promoter for the sp6 RNA polymerase | |  |

**Table S3.** Number of individual sperm assessed for each condition (all replicates combined)

| Parameter | CP (mg/ml) | | | | CHX (mg/ml) | | | | | |
| --- | --- | --- | --- | --- | --- | --- | --- | --- | --- | --- |
|  | Control | 0.1 | 0.5 | 1.0 | Control | 0.1 | 0.5 | 1.0 | 1.5 | 2.0 |
| Motile/Progressively motile sperm | 1163 | 1007 | 924 | 1004 | 912 | 909 | 805 | 785 | 809 | 746 |
| Live sperm (%) | 2203 | 2129 | 2253 | 2178 | 2156 |  |  | 2168 | 2141 | 2181 |
| VCL, VSL, VAP, ALHavg | 870 | 754 | 553 | 428 | 701 | 531 | 443 | 435 |  | 416 |
| fBF, fAWL, P30 | 441 | 387 | 270 | 217 | 336 | 253 | 209 | 225 |  | 201 |

ALHavg: average Amplitude of Lateral Head displacement; fAWL: flagellar arcwavelength; fBF: flagellar Beat Frequency; P30: Power of the first 30 µm of flagellum (1 fW = 1E-15 W); VAP: Average Path Velocity, VCL: Curvilinear Velocity, VSL: Straight Line Velocity.

**Table S4.** Transition list for the MRM analysis of selected proteins 🡪 Excel file

**Table S5.** Influence of mitochondrial (chloramphenicol, CP) and cytoplasmic (cycloheximide, CHX) ribosome inhibitors on sperm parameters, shown through fitting linear mixed-effects models. The fixed effect coefficients for intercept and concentration of inhibitors shown with 95% confidence intervals and number of donors n.

| Parameter | CP | | | | | CHX | | | | |
| --- | --- | --- | --- | --- | --- | --- | --- | --- | --- | --- |
|  | Intercept  (95% CI) | p-value | Concentration  (95% CI) | p-value | n | Intercept  (95% CI) | p-value | Concentration  (95% CI) | p-value | n |
| Motile sperm (%)^†^ | 1.1  (0.80, 1.5) | <0.001^**^ | -0.44  (-0.74, -0.14) | 0.0043^*^ | 8 | 1.2  (0.91, 1.6) | <0.001^**^ | -0.032  (-0.19, 0.12) | 0.68 | 7 |
| Progressively motile sperm (%)^†^ | 0.88  (0.52, 1.2) | <0.001^**^ | -0.80  (-1.2, -0.42) | <0.001^**^ | 8 | 0.93  (0.61, 1.3) | <0.001^**^ | -0.073  (-0.22 0.75) | 0.33 | 7 |
| Live sperm (%)^†^ | 2.0  (1.7, 2.2) | <0.001^**^ | -0.013  (-0.12, 0.099) | 0.82 | 7 | 2.0  (1.7, 2.2) | <0.001^**^ | -0.029  (-0.11, 0.055) | 0.50 | 7 |
| ATP (nM/1 million sperm)^†^ | 650  (570, 720) | <0.001^**^ | -35  (-180, 110) | 0.63 | 9 | 660  (610, 710) | <0.001^**^ | -31  (-80, 19) | 0.22 | 9 |
| Relative densitometry phosphotyrosines/beta-tubulin^†^ | 1.1  (0.81, 1.4) | <0.001^**^ | -0.011  (-0.44, 0.22) | 0.50 | 9 | 1.3  (0.81, 1.8) | <0.001^**^ | -0.15  (-0.34, 0.029) | 0.10 | 7 |
| VCL (µm/s) | 98  (84, 110) | <0.001^**^ | -28  (-41, -14) | <0.001^**^ | 7 | 99  (87, 110) | <0.001^**^ | 0.15  (-3.3, 3.6) | 0.93 | 7 |
| VSL (µm/s) | 58  (48, 68) | <0.001^**^ | -15  (-22, -7.5) | <0.001^**^ | 7 | 52  (42, 62) | <0.001^**^ | -1.9  (-4.1, 0.27) | 0.086 | 7 |
| VAP (µm/s) | 49  (44, 54) | <0.001^**^ | -8.8  (-13, -4.5) | <0.001^**^ | 7 | †† | †† | †† | †† | 7 |
| ALHavg (µm) | 1.9  (1.5, 2.3) | <0.001^**^ | -0.61  (-1.0, -0.21) | 0.0028^*^ | 7 | 1.7  (1.4, 2.1) | <0.001^**^ | 0.021  (-0.070, 0.11) | 0.66 | 7 |
| fBF (µm) | †† | †† | †† | †† | 7 | †† | †† | †† | †† | 7 |
| fAWL (µm) | 15  (14, 16) | <0.001^**^ | -0.20  (-1.3, 0.86) | 0.71 | 7 | 16  (15, 17) | <0.001^**^ | -0.075  (-0.55, 0.40) | 0.75 | 7 |
| P30 (fW) | 6.6  (5.2, 8.1) | <0.001^**^ | -2.5  (-4.2, -0.82) | 0.0037^*^ | 7 | 7.0  (6.0, 8.1) | <0.001^**^ | 0.0064  (-1.3, 1.5) | 0.93 | 7 |

Purified human spermatozoa were incubated for 4 h in a capacitation medium in the absence (control) or presence of CP and CHX. For CP, all parameters were tested at concentrations 0.1, 0.5, and 1.0 mg/ml. For CHX, sperm motility, ATP content, and phosphotyrosine content were tested at concentrations 0.1, 0.5, 1.0, 1.5, and 2.0 mg/ml, vitality was measured at concentrations 1.0, 1.5, and 2.0 mg/ml, and kinematic parameters were tested at 0.1, 0.5, 1.0, and 2.0 mg/ml. For each parameter (P) a linear mixed effects model: P ~ 1 + Concentration + (1 + Concentration | Donor) was fit and the fixed effect coefficient for concentration reported. Models indicated by †† did not converge and the results are therefore not shown. * p-value ≤ 0.05, ** p-value ≤ 0.001. ALHavg: average Amplitude of Lateral Head displacement; fAWL: flagellar arcwavelength; fBF: flagellar Beat Frequency; P30: Power of the first 30 µm of flagellum (1 fW = 1E-15 W); VAP: Average Path Velocity, VCL: Curvilinear Velocity, VSL: Straight Line Velocity.

**Table S6**. Normalized abundance of proteins identified in CHX (Table S6a) and in CP (Table S6b) conditions 🡪 Excel file

**Table S7**. Fold change for each protein identified in CHX (Table S7a) and in CP (Table S7b) conditions 🡪 Excel file

**Table S8**. Raw data from the MRM analysis 🡪 Excel file
